# Gastric inflammatory myofibroblastic tumor: a case report

**DOI:** 10.1186/s40792-024-01844-7

**Published:** 2024-03-15

**Authors:** Taku Hattori, Yutaka Tanizawa, Tadakazu Shimoda, Yusuke Koseki, Kenichiro Furukawa, Keiichi Fujiya, Daisuke Aizawa, Takashi Sugino, Masanori Terashima, Etsuro Bando

**Affiliations:** 1 Divisions of Gastric Surgery, Shizuoka Cancer Center, 1007 Shimonagakubo, Nagaizumi-Cho, Sunto-Gun, Shizuoka, 411-8777 Japan; 2 Divisions of Pathology, Shizuoka Cancer Center, 1007 Shimonagakubo, Nagaizumi-Cho, Sunto-Gun, Shizuoka, 411-8777 Japan

**Keywords:** Inflammatory myofibroblastic tumor, Stomach, Submucosal tumor, Gastric tumor

## Abstract

**Background:**

Inflammatory myofibroblastic tumor (IMT) of the stomach is an uncommon mesenchymal neoplasm. We present a case of gastric submucosal tumor (SMT) where the final diagnosis was IMT.

**Case presentation:**

A 69-year-old man presented with a 24-mm SMT on the posterior wall of the middle third of the stomach that was detected by screening upper gastrointestinal endoscopy. Abdominal contrast-enhanced computed tomography showed that the tumor was well-enhanced. Although endoscopic ultrasonography-guided biopsy was performed, the histological diagnosis was not confirmed preoperatively. Since the tumor was clinically suspected to be a gastrointestinal stromal tumor, we performed gastric wedge resection by laparoscopic–endoscopic cooperative surgery. Pathologically, proliferative spindle cells with a positive reaction for smooth muscle actin, negativity for c-kit, desmin, s-100, CD34, STAT-6, β-catenin and anaplastic lymphoma kinase 1 were identified. Hence, the tumor was finally diagnosed as an IMT originating from the stomach.

**Conclusions:**

When an SMT of the stomach is identified, the possibility of gastric IMT should be considered.

## Background

Inflammatory myofibroblastic tumor (IMT) is classified as an intermediate malignant neoplasm by the World Health Organization Histological Typing of Soft Tissue Tumors [1]. IMT of the stomach is an extremely rare tumor of uncertain etiology with a variety of clinical features, which makes it difficult to diagnose preoperatively. Here, we present a case of primary gastric IMT, along with a review of the relevant literature.

## Case presentation

**Fig. 1:**
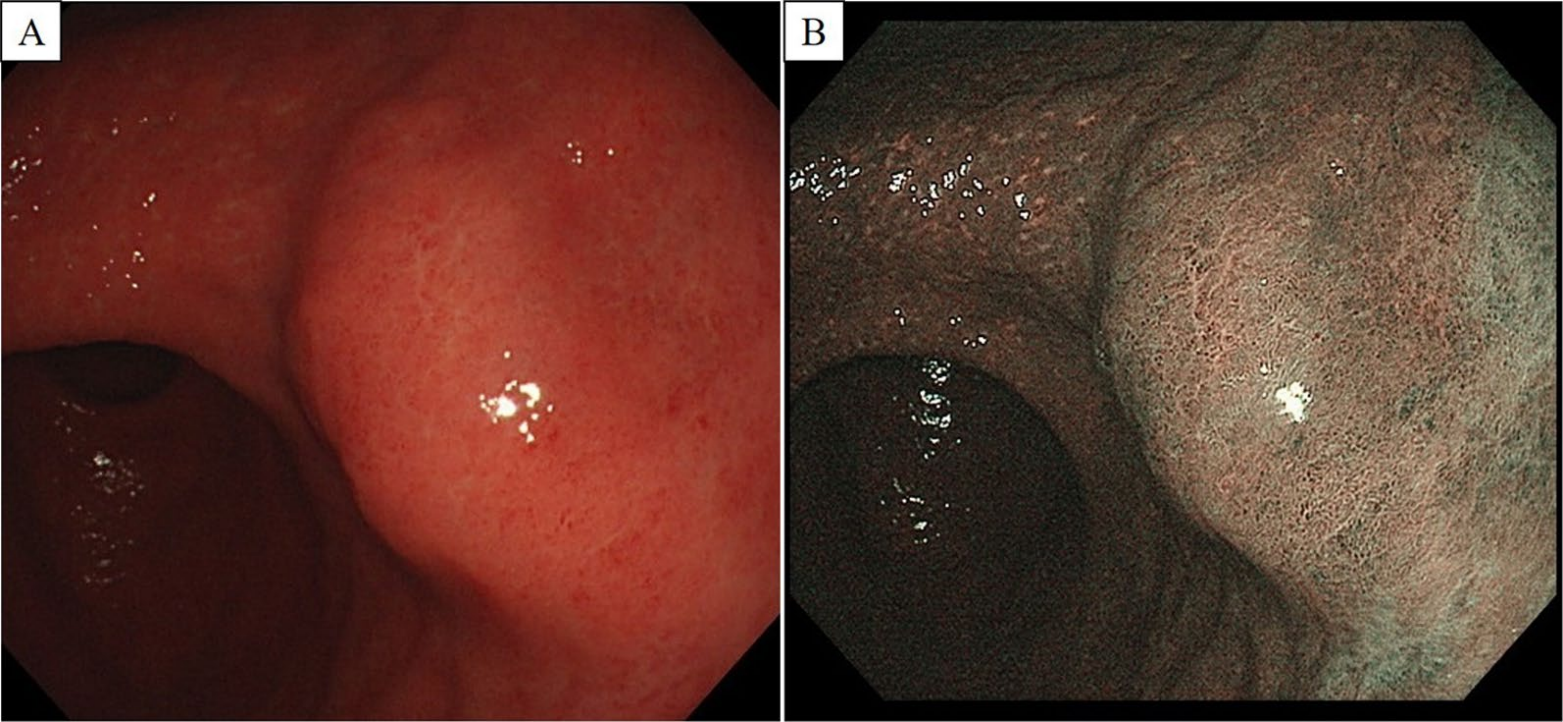
Upper gastrointestinal endoscopy. **A** Conventional endoscopy. **B** Narrow-band imaging endoscopy. Upper gastrointestinal endoscopy revealed the submucosal mass as a flat protrusion on the posterior wall of the middle third of the stomach

**Fig. 2:**
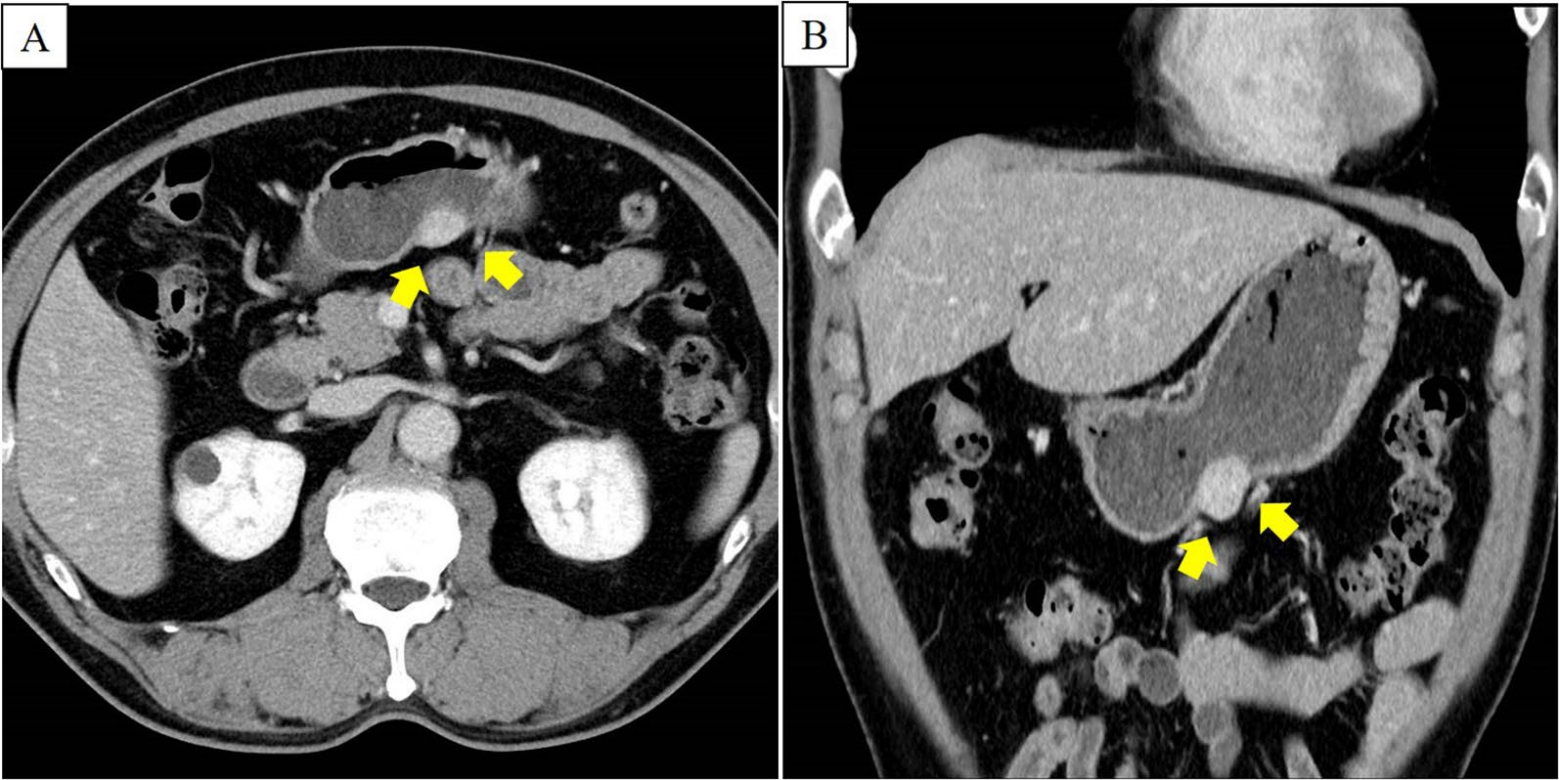
Abdominal enhanced computed tomography. **A** Horizontal view. **B** Coronal view. Abdominal enhanced CT scan showed a contrast-enhanced round mass on the posterior wall of the stomach (yellow arrows), with no evidence of tumor metastasis

**Fig. 3:**
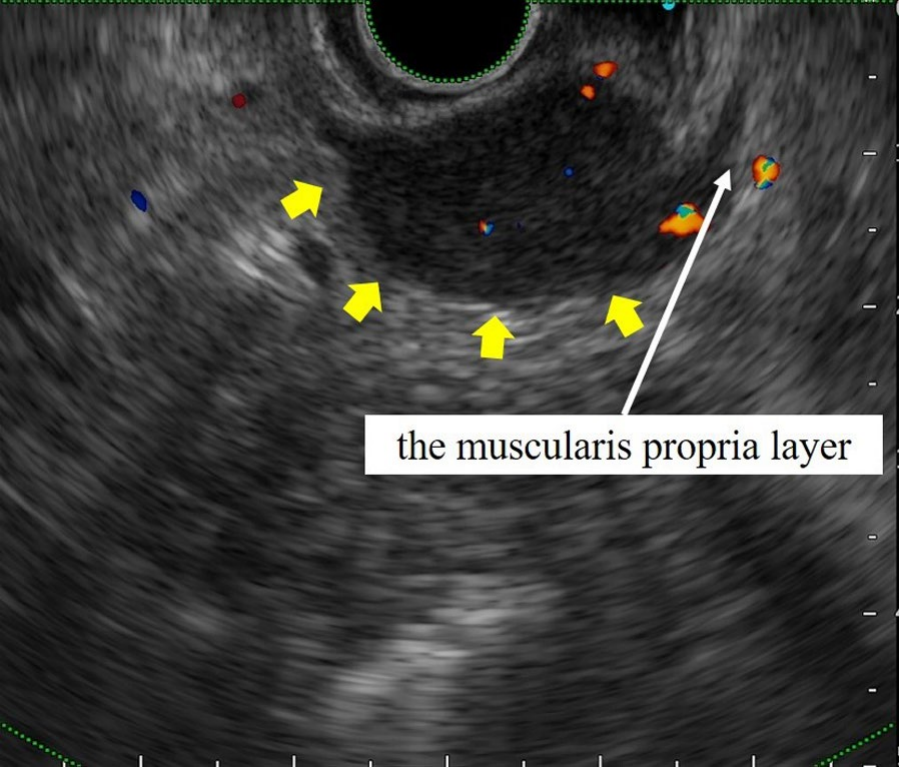
Endoscopic ultrasonography. Endoscopic ultrasonography demonstrated a hypoechoic mass, 22 × 20 mm in diameter (yellow arrows). The white arrows indicate the muscularis propria layer

**Fig. 4:**
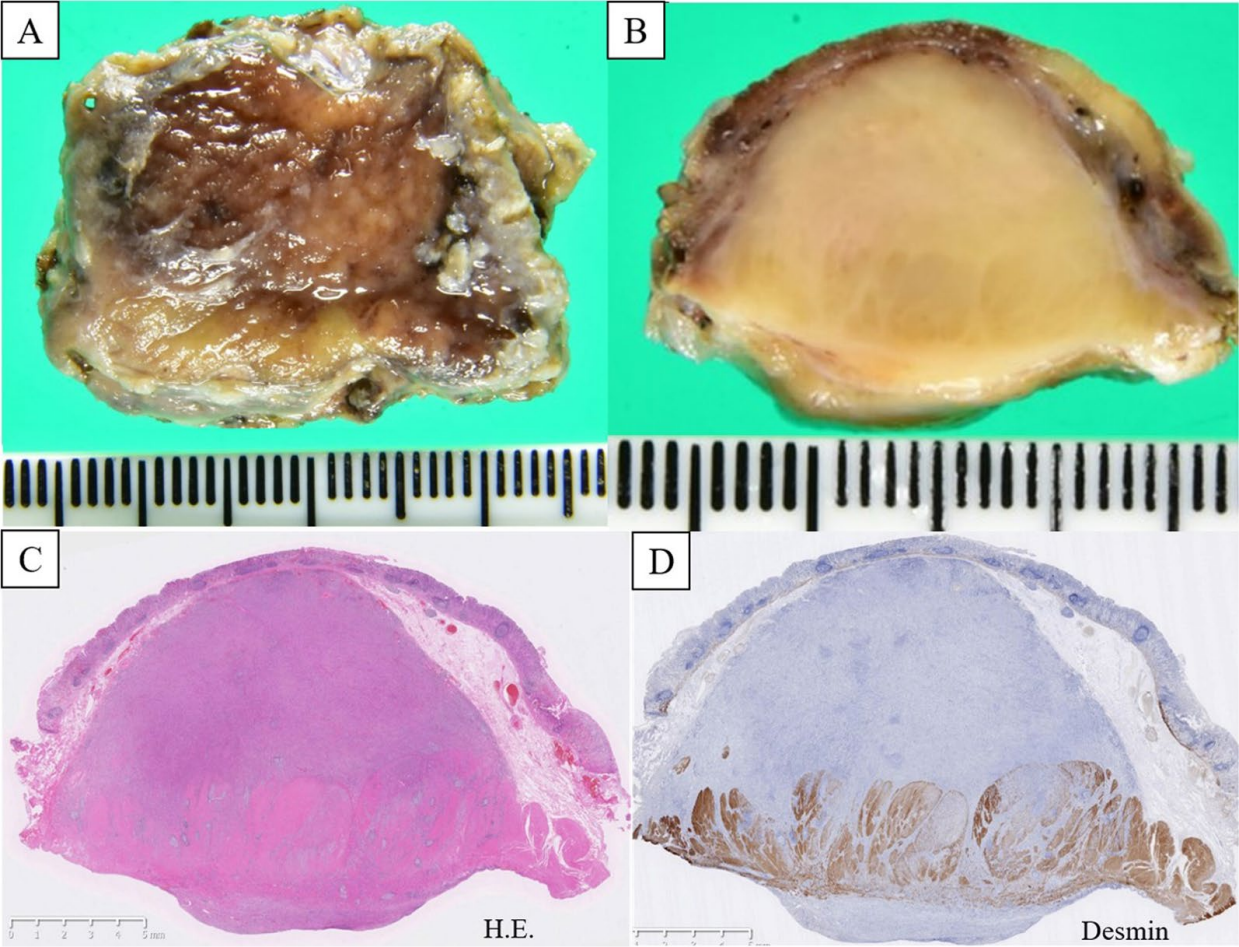
**A**, **B** Macroscopically, the tumor presented as a 15 mm, round nodule, with a yellowish-white cut surface. **C** The tumor consisted of spindle cells spreading from the submucosal layer to the muscularis propria layer (Hematoxylin–eosin staining, × 1). **D** The spindle cells showed negative staining for anti-desmin antigen (× 1)

**Fig. 5:**
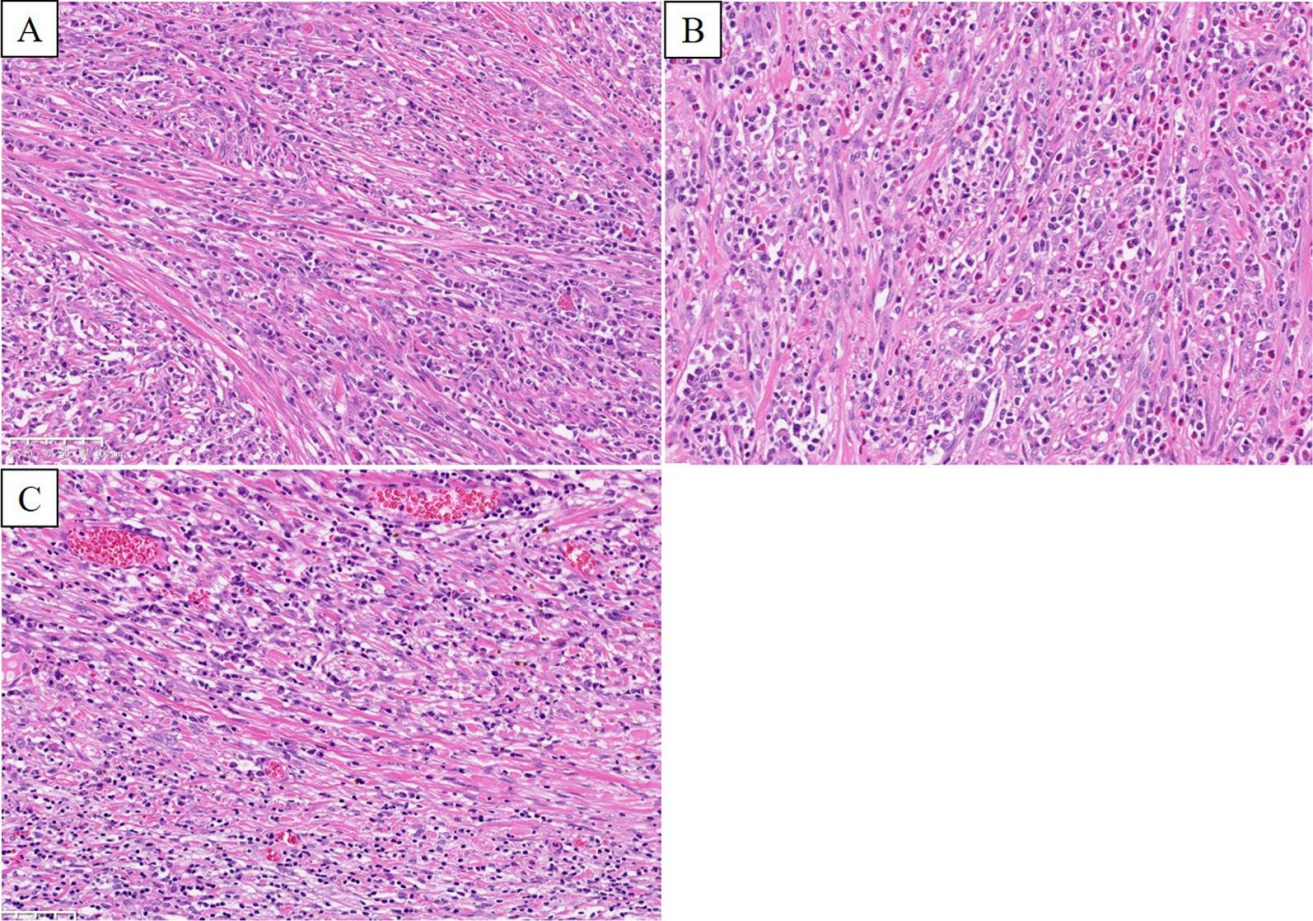
Pathological evaluation of the tumor (Hematoxylin–eosin staining, × 20). **A** The mass was characterized by spindle cell proliferation. **B** The stroma was infiltrated by inflammatory cells. **C** Spindle cell tumors extended into the subserosal layer

**Fig. 6:**
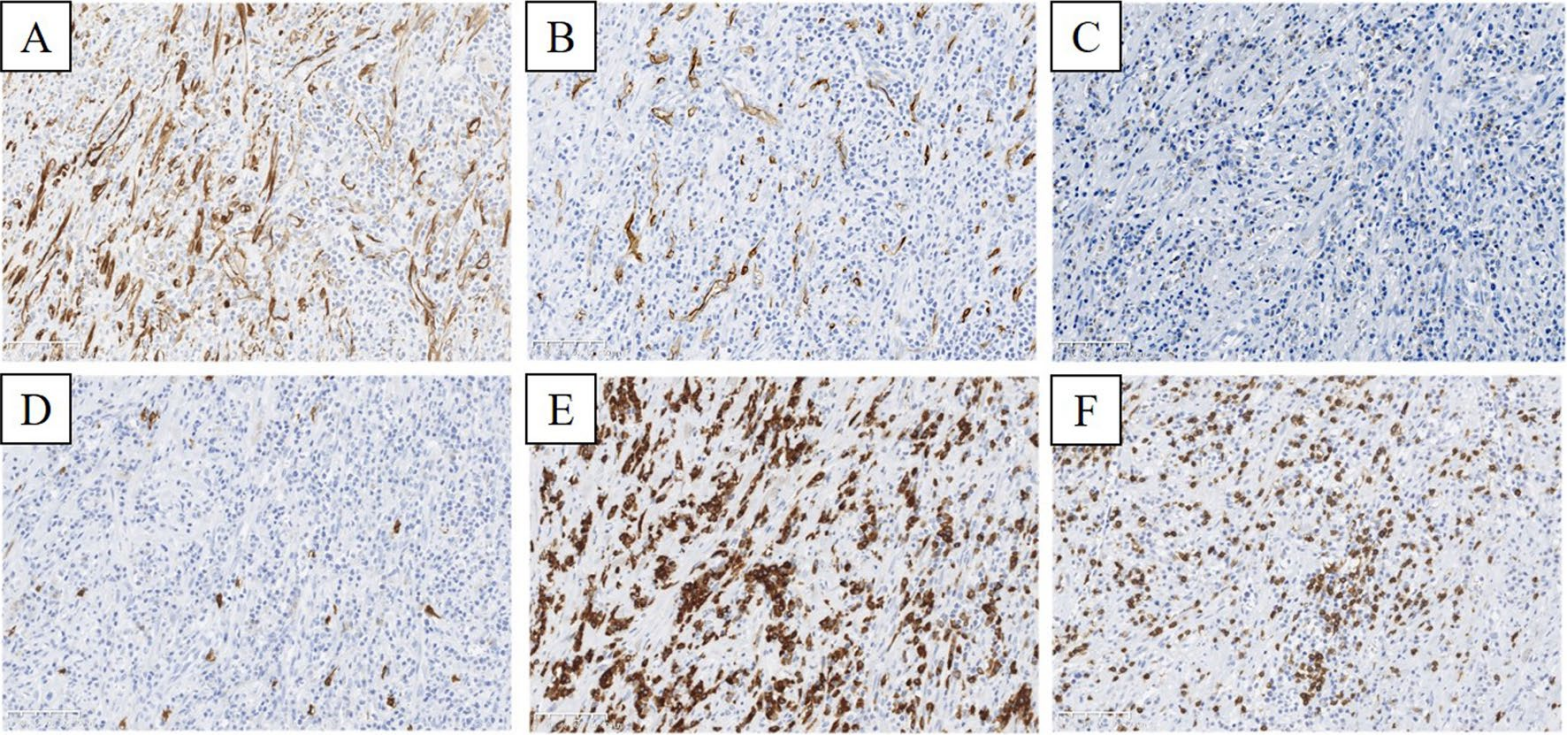
Immunohistochemical staining of the tumor (× 40). **A**, **B** The tumor cells were slightly positive for ASMA (**A**) and CD34 (**B**). **C**, **D** They were completely negative for STAT6 (**C**) and c-kit (**D**). **E**, **F** They were positive for CD79a (**E**) and CD3 (**F**)

A 69-year-old man presented with an asymptomatic gastric submucosal tumor (SMT) that was detected by screening upper gastrointestinal endoscopy at another hospital. Contrast-enhanced chest and abdominal computed tomography (CT) showed a mass on the gastric wall and a tumor in the right lung. Thoracoscopic partial resection of the right lung was first performed for diagnosis and treatment of the lung tumor, and the resected specimen was pathologically suspected as being a metastatic lung tumor from thyroid papillary carcinoma. He was referred to our hospital for further examination and treatment for thyroid cancer and the gastric SMT. Evaluation of the gastric SMT by upper gastrointestinal endoscopy at our hospital revealed a flat protrusion without surface ulceration on the posterior wall of the middle third of the stomach (Fig. 1). Contrast-enhanced abdominal CT demonstrated a well-enhanced solid mass at the greater curvature of the stomach, measuring 22 × 18 mm (Fig. 2). Endoscopic ultrasonography (EUS) showed a hypoechoic mass, 22 mm in diameter, arising from the muscularis propria layer (Fig. 3). Endoscopic ultrasonography-guided fine needle aspiration (FNA) identified spindle cell nests with inflammation of the stomach. Immunohistochemically, the spindle cells were partially positive for alpha smooth muscle actin (ASMA) and desmin, but negative for c-kit, CD34, discovered on GIST-1 (DOG1) and S-100. Although he was diagnosed as having a gastrointestinal tumor of the stomach, the preoperative histological diagnosis was inconclusive. At the same time, contrast-enhanced neck CT showed a nodule-aggregating lesion with calcification in the right lobe of the thyroid grand, and enlarged regional lymph nodes. He was diagnosed with papillary thyroid carcinoma and cervical lymph node metastasis by neck ultrasonography-guided biopsy. Thus, we planned to first perform surgery for the thyroid malignancy, followed by partial gastric resection. Histopathological evaluation of the resected specimen obtained by total thyroidectomy with radical cervical lymph node resection, performed by the department of head and neck surgery at our hospital, confirmed the diagnosis. Subsequently, we performed gastric wedge resection of the gastric SMT by laparoscopic–endoscopic cooperative surgery. Macroscopic evaluation of the gastric tumor showed a circular tumor, measuring 35 × 25 × 15 mm, with a whitish cut surface. The tumor invaded the muscular layer of the gastric wall, and the surgical margin was negative (Fig. 4). Microscopic examination revealed well-circumscribed, spindle-shaped tumor cells, consisting of fibroblasts, myofibroblasts and eosinophils, accompanied by myxoid changes and collagen fibers mainly in the stroma, and identified in the gastric wall from the lamina propria to the intrinsic muscularis (Fig. 5). There was no atypia, necrosis, nuclear fission, or calcification. On immunohistochemical evaluation, the spindle cells showed positive immunoreactivity for ASMA and partial positivity for calponin, but negativity for anaplastic lymphoma kinase-1 (ALK-1), S-100, desmin D33, c-kit, CD34, DOG1, CD56, and β-catenin, while the IgG4/IgG ratio was 10–20% and Ki-67 labeling index was 10–20% (Fig. 6). The final diagnosis was consistent with an IMT originating from the stomach. The patient was uneventfully discharged from the hospital on postoperative day 7, and no recurrence of IMT was observed on CT at 29 months after surgery.

## Discussion

IMT was first reported in two cases of benign pulmonary spindle cell tumors by Brunn [2] in 1939. It usually involves the lung and occurs in children and young adults [3]. It is currently classified as an intermediate neoplasm according to the World Health Organization Histological Typing of Soft Tissue Tumors [1]. Although its etiology and pathogenesis are still controversial, the development of IMT has been hypothesized to be affected by many causes, such as infection, trauma, surgery, autoimmunity, and chromosomal variation of the ALK gene [4]. Distant metastasis of this tumor is rare, occurring in 5% of cases. The most common sites of metastases are the lung and brain, followed by the liver and bone [5].

Making an accurate preoperative diagnosis of IMT is difficult. Patients with IMT sometimes present anemia on laboratory analyses, although there are no characteristic findings for IMT on CT and EUS. Hence, most IMT cases require surgical exploration to obtain an accurate microscopic diagnosis [4]. Complete resection has been proposed as the treatment of this tumor since residual tumor can cause local recurrence. However, the optimal surgical extent and approach are unclear [1, 3]. The recurrence rate after resection is ~ 25% [1], with most cases showing local recurrence.

Pathologically, IMT is composed of myofibroblastic spindle cells accompanied by an inflammatory infiltrate of plasma cells, lymphocytes and eosinophils, and usually occurs in the soft tissue and viscera. Immunohistochemically, the spindle cells are reactive against antibodies to vimentin and ASMA [6]. Furthermore, rearrangements of the ALK gene on chromosome 2p23 are suggested in the pathogenesis of IMT [7]. Three architectural patterns for IMT have been described: myxoid hypocellular pattern; a cellular fascicular or nested pattern with variable amounts of myxoid stroma; and a sclerotic, hyalinized pattern with minimal myxoid stroma. However, these patterns are often admixed in a single tumor [3]. There are no requirements for the diagnosis on pathological examination. It is only after other mesenchymal tumors have been ruled out that a definitive diagnosis is made.

Since only few reviews have provided detailed reports of the clinicopathological features of gastric IMT, we conducted a review of 41 clinical cases associated with gastric IMT that were resected surgically or endoscopically by performing a search of the PubMed database. The details of these cases, including the present case, are shown in Tables 1 and 2 [8–42].

**Table 1:** Clinical findings in 41 cases of gastric IMT

Clinical findings	No. of patients	
	(*n* = 41)	[%]
Age (y.o)		
Range	0–80	
Median	42	
Sex		
Male	17	[42]
Female	24	[59]
Symptom		
Abdominal pain	20	[49]
Anemia	14	[34]
Body weight loss	10	[24]
Abdominal discomfort	4	[10]
Gastrointestinal bleeding	4	[10]
Malaise	4	[10]
Fever (BT > 37.0 ℃)	2	[5]
Gastroesophageal reflux	2	[5]
Dysphagia	1	[2]
Coma	1	[2]
Syncope	1	[2]
No complaint	1	[2]
NA	3	[7]
Preoperative diagnosis		
IMT	2	[5]
GIST	8	[20]
Ectopic pancreas	2	[5]
Gastric polyp	1	[2]
Gastric hemangioma	1	[2]
Gastric leiomyoma	1	[2]
Gastric cancer	1	[2]
Gastric teratoma	1	[2]
Gastric myoma	1	[2]
Mucinous cystic neoplasm	1	[2]
Adrenal neuroblastoma	1	[2]
NA	21	
Site of synchronous metastasis		
Greater omentum	1	[2]
Small intestine	1	[2]
Treatment		
Partial gastrectomy	23	[56]
Distal gastrectomy	6	[15]
Proximal gastrectomy	6	[15]
Total gastrectomy	3	[7]
Gastrectomy with other organ resection	8	[20]
EMR	2	[5]
ESD	1	[2]
Tumor size from the specimen (mm)		
Range	15–220	
Median	55	

*NA* not available, *Hb* hemoglobin, *BT* body temperature, *EMR* endoscopic mucosal resection, *ESD* endoscopic submucosal dissection

**Table 2:** Endoscopic and CT imaging findings in 41 cases of gastric IMT

Characteristics of endoscopy and CT image	No. of patients	
	(*n* = 41)	[%]
Tumor localization (length axis)		
Cardia	6	[15]
Body	29	[71]
Pylorus	3	[7]
NA	3	[7]
Tumor localization (short axis)		
Greater curvature	9	[22]
Lesser curvature	7	[17]
Anterior wall	5	[12]
Posterior wall	13	[32]
NA	7	[17]
Direction of tumor growth		
Intraluminally	19	[46]
Extraluminally	11	[27]
Intra and extraluminally	9	[22]
NA	2	[5]
Site of infiltration		
Spleen	3	[7]
Pancreas	2	[5]
Left diaphragm	1	[2]
Liver	1	[2]
Esophagus	1	[2]
Lung	1	[2]
CT enhancement		
Heterogeneously enhanced	12	[29]
Homogeneously enhanced	4	[10]
None or hypointense	6	[15]
NA	19	[46]
Tumor with calcification		
Positive	4	[10]
Negative	30	[73]
NA	7	[17]
Tumor with ulceration		
Positive	16	[39]
Negative	20	[49]
NA	5	[12]

Median patient age was 42 years, and the number of women was 24 (58.5%). In terms of clinical manifestations, abdominal pain was recognized in 20 cases (48.8%), followed by anemia, weight loss, nausea, and vomiting. Tumors were mostly located in the body of the stomach (in 29 cases, 70.7%), and mainly on the posterior wall, followed by the greater and lesser curvatures. Synchronous metastases of seed nodules in the small intestine and greater omentum were seen in only one case, and six cases (14.6%) exhibited direct infiltration of the spleen, pancreas, left diaphragm, esophagus, and hilum of the left lung. Although biopsy was performed preoperatively in 16 cases (39.0%), only two cases (4.9%) were diagnosed as gastric IMT, both of which were diagnosed in the same institution and had a high degree of invasion. Most cases were misdiagnosed as gastrointestinal stromal tumor (GIST). Eighteen cases had iodine-induced contrast effects on enhanced CT imaging. The median tumor size was 55 mm, and the intraluminally growing type was recognized in 20 cases (48.8%). Tumors with calcification were recognized in four cases (9.8%), and ulceration was seen in 16 cases (39.0%).

**Fig. 7:**
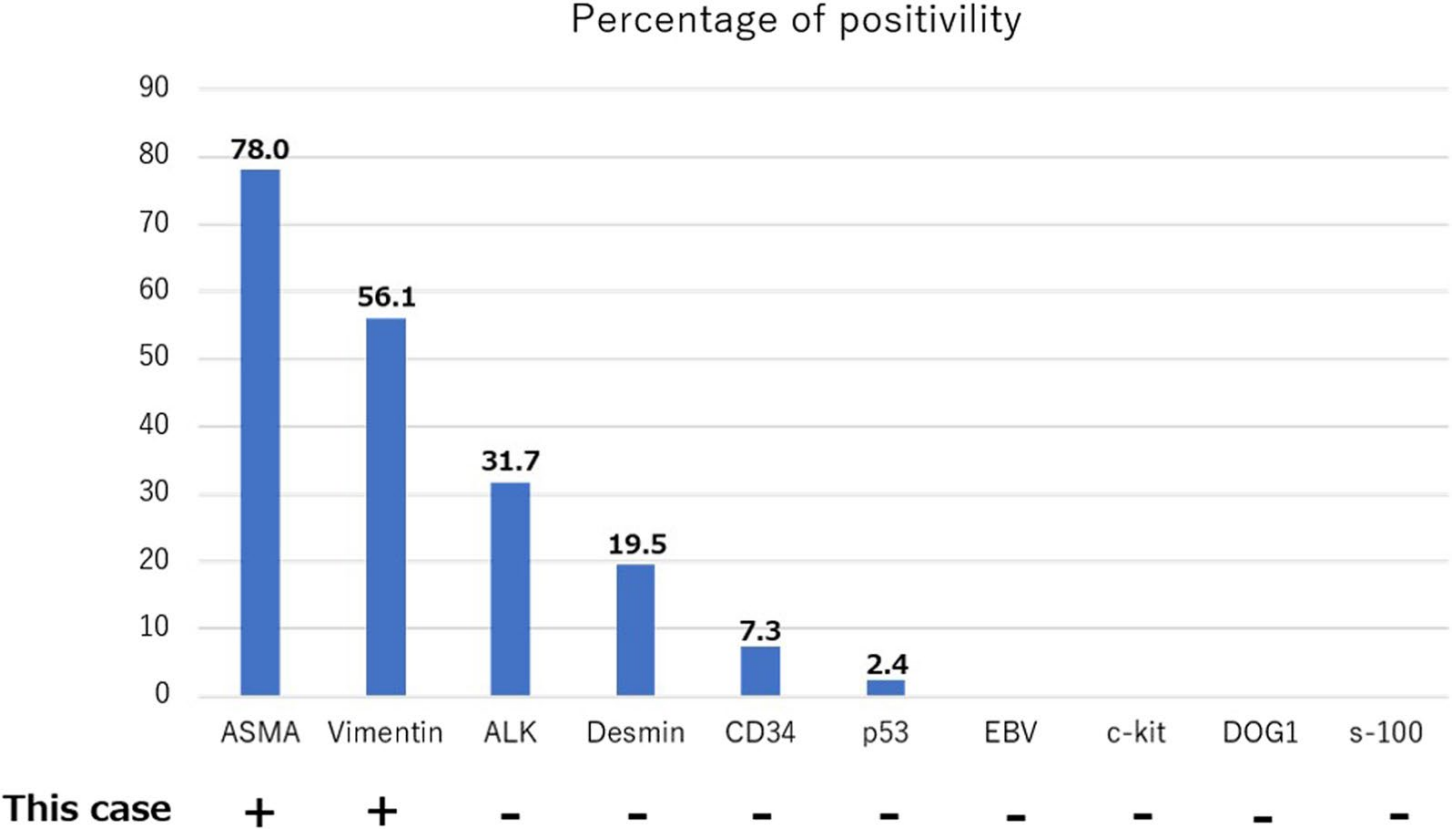
Immunohistochemical findings in 41 cases of gastric IMT. *EBV* Epstein–Barr virus, *ALK* anaplastic lymphoma kinase, *ASMA* alpha smooth muscle actin, *DOG* discovered on GIST, +:  positive, −: negative

Partial gastrectomy was performed in 24 cases (58.5%), distal gastrectomy and proximal gastrectomy in six cases each (14.6%), and total gastrectomy in three cases (7.3%). In all cases, spindle tumor cells were identified immunohistochemically. In addition, the cells were positive for ALK in 13 cases (31.7%), for ASMA in 32 cases (78.0%), and for vimentin in 23 cases (56.1%) (Fig. 7). On the other hand, the tumors stained negatively for Epstein–Barr virus (EBV)-determined nuclear antigen, latent membrane protein-1 (LMP-1), and EBV-encoded small RNA in all six of the cases evaluated. In contrast with GIST, none of these tumors was positive for c-kit or DOG1, and fewer of them stained positive for CD34 (3 cases, 7.3%). Recurrence was observed in only two cases (4.9%), the site of which was the remnant stomach and the small intestine. The above observations showed that gastric IMT has a lot in common with IMT occurring in other organs. However, there is no specific clinical feature to identify IMT from among gastric SMTs.

In our case, the patient was relatively older, and the tumor was smaller compared to previous reports. In addition, our case had no symptoms, as was seen in only one case in the previous literature. We assumed that our case had no symptoms because of the small tumor size, which prevented from being identified earlier.

FNA is usually performed in the preoperative diagnostic work-up of gastric SMT. Only the cellular components are extracted by FNA, as opposed to the entire tissue by surgical specimen. For this reason, it is often difficult to determine tumor or non-tumor and to reach a definitive diagnosis, especially for mesenchymal tumors. Surgical treatment for diagnostic purposes was selected because biopsies had already been done for three specimens, and Japanese practice guidelines for GIST state that an SMT of 2–5 cm in size is a relative indication for resection [43, 44].

Positive surgical margins are the most common causes of local recurrence, which is the most common type of recurrence, according to previous IMT reports [1]. Although several reports suggested that the anatomic site of IMT is associated with the recurrence rate [1], there are no detailed reports of postoperative recurrence of primary gastric IMT. In the present review, two cases showed recurrence. One patient had local recurrence after partial gastrectomy, splenectomy, and omentectomy with a positive resection margin on the peritoneum. Another patient had a small bowel stricture due to peritoneal recurrence after proximal gastrectomy, partial diaphragmectomy, and partial left pneumonectomy. Although ALK reactivity was found to be associated with local recurrence [2], our review did not find evidence for this connection. In addition, though 26 of the 41 patients (63.4%) underwent partial resection of the stomach, not a single recurrence occurred at the remnant stomach. Consequently, it was considered unlikely that local excision would lead to a positive resection margin and further local recurrence.

As in other cases, partial gastrectomy was performed for diagnosis and treatment. In particular, laparoscopic–endoscopic cooperative surgery is beneficial because it allows evaluation of tumor progression and safe resection of the margin of the tumor, particularly in cases that are undiagnosed preoperatively.

## Conclusions

Gastric IMT is a rare mesenchymal tumor with uncertain physical and inspection findings. Our experience suggests that IMT should be considered in the differential diagnosis of gastric SMTs, which may contribute to the safe and complete resection.

## Data Availability

The data supporting the conclusions of this case report are included within the article.

## Abbreviations

IMT: Inflammatory myofibroblastic tumor
SMT: Submucosal tumor
CT: Computed tomography
EUS: Endoscopic ultrasonography
FNA: Fine needle aspiration
ASMA: Alpha smooth muscle actin
DOG: Discovered on GIST
ALK: Anaplastic lymphoma kinase
EBV: Epstein–Barr virus
NA: Not available
Hb: Hemoglobin
BT: Body temperature
EMR: Endoscopic mucosal resection
ESD: Endoscopic submucosal dissection
